# Combination Antitumor Effect of Sorafenib via Calcium-Dependent Deactivation of Focal Adhesion Kinase Targeting Colorectal Cancer Cells

**DOI:** 10.3390/molecules25225299

**Published:** 2020-11-13

**Authors:** Keun-Yeong Jeong, Minhee Park, Jae-Jun Sim, Hwan Mook Kim

**Affiliations:** 1 R&D Center, Metimedi Pharmaceuticals, 263, Central-ro, Yeonsu-gu, Incheon 22006, Korea; pmh1880@hanmail.net (M.P.); genesis0804@hanmail.net (J.-J.S.); 2 Gachon Institute of Pharmaceutical Science, Gachon University, 191, Hambangmoe-ro, Yeonsu-gu, Incheon 21936, Korea

**Keywords:** colorectal cancer, sorafenib, lactate calcium salt, focal adhesion kinase, antitumor effect

## Abstract

Sorafenib has been recently used for the treatment of patients with advanced colorectal cancer (CRC) and is recognized for its therapeutic value. However, the continuous use of sorafenib may cause resistance in the treatment of cancer patients. In this study, we investigated whether sorafenib exerts an enhanced anticancer effect on CRC cells via the calcium-mediated deactivation of the focal adhesion kinase (FAK) signaling pathways. The appropriate dose of sorafenib and lactate calcium salt (CaLa) for a combination treatment were determined using 3-(4,5-dimethylthiazol-2-yl)-2,5-diphenyltetrazolium bromide assays. Then, cell cycle analysis was performed following treatment with 2.5 μM sorafenib and/or 2.5 mM CaLa. CRC cells were found to be in the G1 phase by sorafenib treatment, and they accumulated in the sub-G1 phase with CaLa treatment. Western blots and enzyme-linked immunosorbent assays were performed to analyze the elements of the recombinant activated factor (RAF) and focal adhesion kinase (FAK) signaling cascades. Sorafenib-inhibited RAF-dependent signaling in CRC cells, however, either did not affect the expression of Akt or increased it. As the upstream signaling of FAK was suppressed by CaLa, we observed that the expression of the sub-signaling phospho (p) AKT and p-mammalian target of rapamycin was also suppressed. Treatment with a combination of sorafenib and CaLa enhanced the antitumor activity of CRC cells. The % viability of CRC cells was significantly decreased compared to the single treatment with sorafenib or CaLa, and the accumulation of Sub G1 of CRC cells was clearly confirmed. The migration ability of CRC cells was significantly reduced. The findings of this study indicate that sorafenib will show further improved antitumor efficacy against CRC due to overcoming resistance through the use of CaLa.

## 1. Introduction

Many tyrosine kinase receptors are located upstream of mitogen-activated protein kinase (MAPK) pathways in colorectal cancer (CRC) cells, and the MAPK signaling pathway was found to be related to oncogenic processes [1]. The function of recombinant activated factor (RAF) family kinases in CRC cells is known to play a role in proliferation, differentiation, and survival [2]. Sorafenib is an oral kinase inhibitor that has activity in patients with advanced solid tumors, and it is the most successful multi-kinase inhibitor targeting the RAF/mitogen-activated protein kinase (MEK)/extracellular signal-regulated kinase (ERK) pathway [1,3]. Therefore, sorafenib can also be expected to be effective for the treatment of patients with advanced CRC. The excellence of sorafenib as a targeted therapy for CRC was verified through non-clinical and clinical studies [4,5,6,7].

As a single agent, sorafenib can decrease the viability of human CRC cells or reduce carcinoembryonic antigens. The antitumor effect of sorafenib in combination with erlotinib or cetuximab on human CRC cells was also investigated [4,5]. In patients with metastatic CRC, sorafenib has been found to increase the median progression-free survival and overall survival when single-treated or combined with an anti-angiogenic agent, such as bevacizumab [6,7]. However, sorafenib may cause resistance, which has become an obstacle in its continuous use in cancer patients. As primary resistance, the abnormal activation of epithelial growth factor receptor (EGFR) and overexpression of its ligands due to the genetic heterogeneity of cancer was reported to lower the antitumor effect of sorafenib [8]. The phosphoinositide 3-kinases (PI3K)/Akt pathway regulates many molecules involved in most aspects of cancer growth and cross-talks with the RAF/MEK/ERK pathway [9]. Therefore, in acquired resistance, the expression of the phosphorylated PI3K/Akt pathway is maintained by sorafenib, and downstream factors, such as mammalian target of rapamycin (mTOR), are activated to act as a potential compensation mechanism [3,9].

Focal adhesion kinase (FAK) is a functioning upstream activator of PI3K and stimulates cancer cell proliferation and migration via the Akt-dependent pathway [10]. FAK promotes phosphorylation of the p85 subunit of PI3K and then indirectly interacts with a collaborative companion protein that can bind to Akt to form a complex [10,11]. It is well established that CRC cells increase FAK expression in the early stages of carcinogenesis [11]. The upregulation of FAK promotes the adhesive properties, survival, and local invasion of CRC cells [11]. Therefore, targeting FAK would be one way to overcome the acquired resistance caused by sorafenib, and an enhanced therapeutic effect on CRC can be expected simultaneously. Our recent study demonstrated that a sustained calcium influx can destroy the constituents of the focal contacts of FAK subject to downregulation of the steroid receptor coactivator (Src), which could involve the inhibition of epithelial-mesenchymal transition signaling, leading to significant suppression of CRC cell invasion and motility [12,13].

In the present study, we investigated whether calcium-mediated deactivation of FAK led to the inhibition of Akt signaling, bypassing the inhibitory pathway of RAF family kinases by sorafenib, and resulting in an enhanced antitumor effect on CRC cells.

## 2. Results

### 2.1. Gradual Antitumor Effect on CRC Cells by the Dose-Dependent Sorafenib Treatment

**Figure 1:**
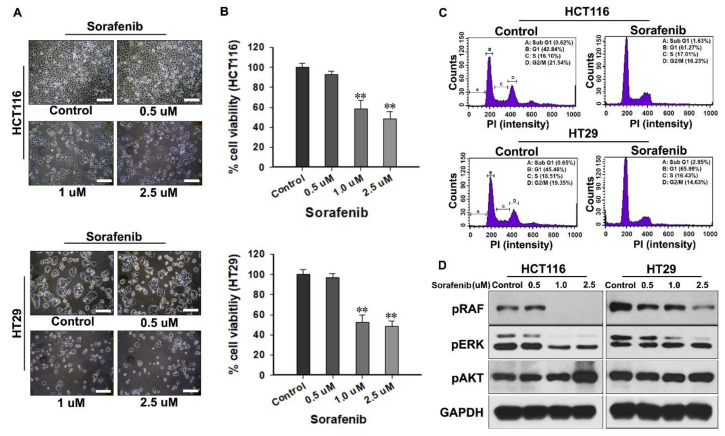
The antitumor effect on colorectal cancer (CRC) cells treated with different concentrations of sorafenib. (**A**) Morphological changes in CRC cells were observed using an optical microscope. Scale bars: 100 μm. Enlarged pictures are provided in Supplementary Figure S2. (**B**) Quantitative analysis of cell viability following treatment of CRC cells with increased concentrations of sorafenib. (**C**) Representative cell cycle profiles of the control and 2.5 μM sorafenib-treated CRC cells. (**D**) Immunoblots showing the suppressive effect of sorafenib on protein expression of phosphor(p) recombinant activated factor (RAF)/p extracellular signal-regulated kinase (ERK) but not for pAkt. Each blot was re-probed with anti-glyceraldehyde-3-phosphate dehydrogenase (GAPDH) antibody to ensure equal protein loading. All results were validated based on the quintuplicate analysis. ** *p* < 0.001 vs. Control. Results are mean ± standard deviation (SD).

The appropriate in vitro applicable concentration of sorafenib for CRC was determined, and the form of the anticancer mechanism was confirmed (Figure 1). The lowest concentration of sorafenib was determined to be 0.5 μM based on previous studies, and the applicable concentration was gradually increased by about two times per group to a maximum of 2.5 μM. The morphological changes in CRC cells were remarkable following the increase in the concentration of sorafenib. After approximately 72 h, the proliferation of CRC cells in the control increased rapidly, whereas in the sorafenib-treated group, the proliferation of CRC cells was delayed and was dependent on the increase in the concentration of sorafenib (Figure 1A). In quantitative analysis, when the viability of the control group was defined as 100%, the viability of CRC cells at concentrations of 0.5, 1.0, and 2.5 μM decreased to 92.8 ± 3.2, 58.1 ± 8.9, and 48.4 ± 7.8% in HCT116 and to 96.8 ± 3.5, 52.1 ± 7.4, and 48.6 ± 5.3% in HT29, respectively (Figure 1B).

Cell cycle analysis was performed in the group treated with 2.5 μM sorafenib, which showed the lowest % cell viability. After 72 h of treatment with sorafenib, most CRC cells were found to be in the G1 phase, while some were found to be partly in the sub-G1 phase (Figure 1C). The expression of phospho-(p) RAF/p extracellular signal-regulated kinase (ERK) gradually decreased with the increase in the concentration of sorafenib, reflecting the intrinsic mechanism of sorafenib. However, the expression of pAkt increased in a collinear trend with an increasing concentration of sorafenib (Figure 1D).

### 2.2. Gradual Dose-Dependent Antitumor Effect of CaLa Treatment on CRC Cells

**Figure 2:**
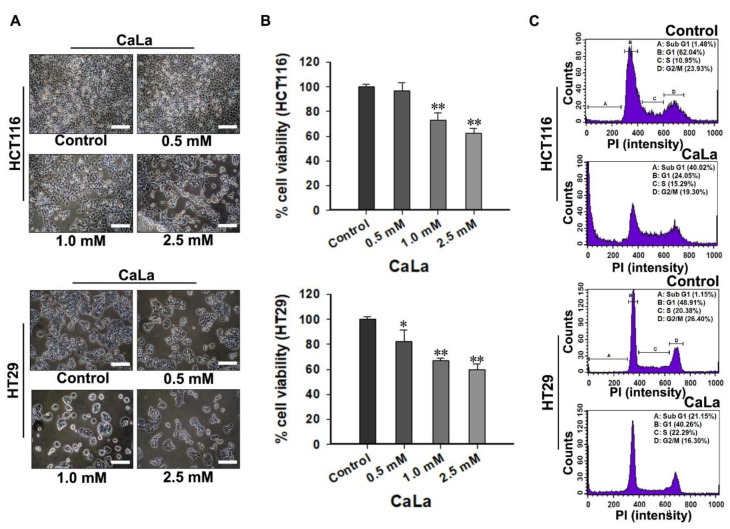
Antitumor effect of lactate calcium salt (CaLa) on colorectal cancer (CRC) cells to determine the appropriate concentration of CaLa in combination with sorafenib. (**A**) Morphological changes in CRC cells were observed using an optical microscope. Scale bars: 100 μm. Enlarged pictures are provided in Supplementary Figure S3. (**B**) Quantitative analysis of the cell viability following treatment of CRC cells with increased concentrations of CaLa. (**C**) Representative cell cycle profiles of the control and 2.5 mM CaLa-treated CRC cells. All results were validated based on quintuplicate analysis. * *p* < 0.05 and ** *p* < 0.001 vs. Control. Results are mean ± SD.

To determine the appropriate concentration of CaLa in combination with sorafenib, CRC cells were treated with increasing concentrations of CaLa. Based on our previous study, 0.5 mM CaLa was set as the starting concentration and was increased up to 2.5 mM to investigate a concentration approaching the lethal dose 50 (Figure 2). In the morphological analysis, CaLa showed gradual inhibition of CRC cell proliferation with some floating cells or remnants of lysis cells observed at the maximum dose of 2.5 mM (Figure 2A). In quantitative analysis, when the viability of the control group was defined as 100%, the viability of CRC cells at concentrations of 0.5, 1.0, and 2.5 mM decreased to 96.9% ± 6.2%, 73.1% ± 5.6%, and 62.4% ± 3.8% in HCT116 and to 81.9% ± 3.5%, 67.1% ± 2.1%, and 59.4% ± 4.9% in HT29, respectively (Figure 2B). Cell cycle analysis performed in the group treated with 2.5 mM CaLa indicated the accumulation of CRC cells in the sub-G1 phase of the cell cycle after 96 h treatment with CaLa (Figure 2C).

### 2.3. Inhibition of FAK-Dependent Downstream Signaling by the Calcium Influx

**Figure 3:**
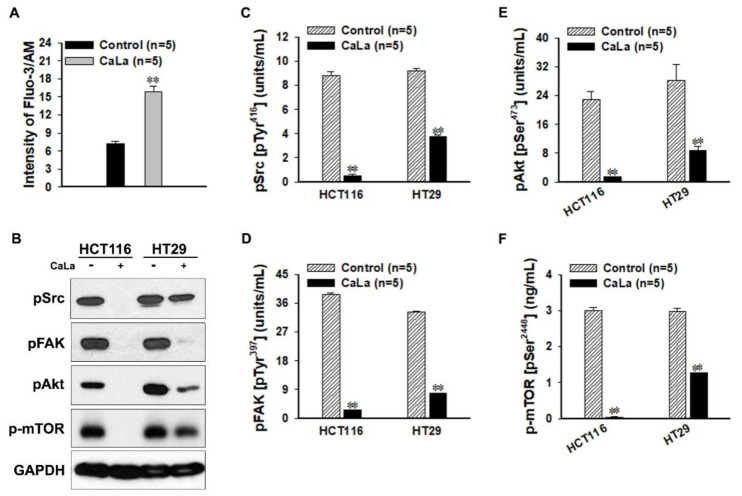
Confirmation of the protein expression of phospho-focal adhesion kinase (pFAK) and the sub-signaling pathways following deactivation of the calcium-dependent steroid receptor coactivator (Src). (**A**) Lactate calcium salt (CaLa)-induced increase in the intracellular calcium influx. (**B**) Immunoblot analysis of pFAK and the sub-signaling pathways in CRC cells. (**C**–**F**) Enzyme-linked immunosorbent assay (ELISA) assay and quantitative analysis for phospho-(p) Src [pTyr^416^], pFAK [pTyr^397^], pAkt [pSer^473^], and p-mammalian target of rapamycin (mTOR) [pSer^2448^] sensitivity in CRC cells. ** *p* < 0.001 vs. Control. Results are mean ± SD.

Next, we confirmed whether treatment with CaLa at the concentration determined in Figure 2 could have a sufficient effect on the FAK-dependent sub-signaling pathway targeting CRC cells (Figure 3). After 96 h of treatment with 2.5 mM CaLa, the concentration of calcium in CRC cells was significantly increased compared to that in the control group (Figure 3A). The expression of pFAK, pAkt, p-mTOR, and pSrc in the lysate of cultured CRC cells was determined qualitatively through immunoblotting, and the expression of all factors in the group to which calcium influx was observed, clearly showed a tendency to decrease (Figure 3B). The quantitative analysis of each factor was compared by analyzing the sensitivity of the activated phospho-form. Phosphorylation of the Tyr^416^ residue in pSrc was observed, and the sensitivity of the group into which calcium was introduced decreased to approximately 85% and 56% in HCT116 and HT29, respectively, compared to the control (Figure 3C). The sensitivity of the phosphorylated form of pFAK at the Tyr^397^ residue was decreased to approximately 93% and 77% in HCT116 and HT29, respectively, compared to the control (Figure 3D). Figure 3E,F show the results for the expression of the sub-signaling pathways that were inhibited by the decrease in the pFAK level. Compared to the control, the pAkt [pSer^473^] and p-mTOR [pSer^2448^] levels were decreased to about 94% and 69% and to about 89% and 58% in HCT116 and HT29, respectively (Figure 3E,F).

### 2.4. Enhanced Antitumor Effect of Sorafenib on CRC Cells by the Calcium Influx

**Figure 4:**
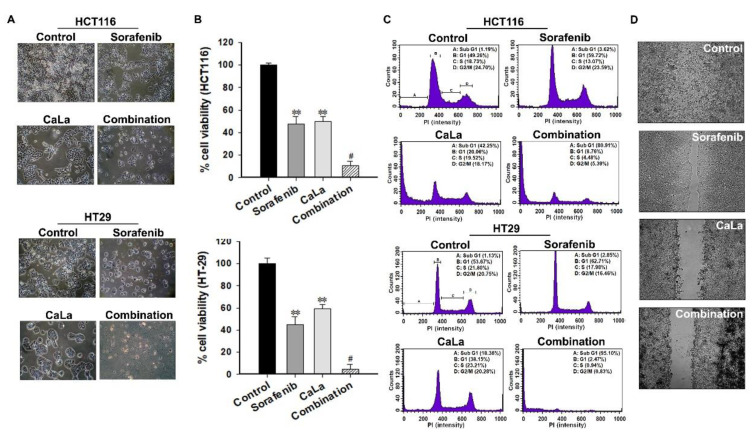
Confirmation of the enhanced anticancer effect of sorafenib through the calcium influx on colorectal cancer (CRC) cells. (**A**) Morphological changes in CRC cells were observed using an optical microscope. Scale bars: 100 μm. Enlarged pictures are provided in Supplementary Figure S4. (**B**) Quantitative analysis of the cell viability following treatment with the combination of sorafenib and lactate calcium salt (CaLa). (**C**) Representative cell cycle profiles of the control, 2.5 μM sorafenib, 2.5 mM CaLa, and combination-treated CRC cells. (**D**) A scratch wound healing assay was performed to determine the horizontal migration ability of CRC cells. All results were validated based on the quintuplicate analysis. ** *p* < 0.001 vs. Control and # *p* < 0.001 vs. sorafenib and CaLa. Results are mean ± SD.

As there was a clear possibility of supplementing the intrinsic mechanism of sorafenib through deactivation of the pFAK and sub-signaling pathways following calcium influx, the combination of sorafenib and CaLa was applied to CRC cells to confirm an enhanced anticancer efficacy (Figure 4). In the morphological analysis, the proliferation of CRC cells in the combination group was significantly suppressed compared to that in the single treatment group, and a large number of death cells or severe remnants of lysis cells were observed (Figure 4A). In quantitative analysis, when the viability of the control group was defined as 100%, the viability of CRC cells in the combination group was decreased to 10.6% ± 4.1% in HCT116 and 4.59% ± 3.6% in HT29, while the viability was 47.5% ± 4.1% (HCT116) and 44.8% ± 6.9% (HT29) in the sorafenib-treated group and 49.9% ± 4.2% (HCT116) and 59.3% ± 3.7% (HT29) in the CaLa-treated group (Figure 4B).

After 96 h of treatment with the combination, the results of cell cycle analysis indicated that most of the CRC cells (80.91% in HCT116 and 95.1% in HT29) were accumulated in the sub-G1 phase. In the sorafenib treatment group, G1 arrest was induced with few cells accumulated in the sub-G1 phase (3.62% in HCT116 and 2.85% in HT29). The percentage of cell accumulation in the sub-G1 phase in the CaLa-treated group was 42.55% and 18.36% in HCT116 and HT29, respectively (Figure 4C). In the evaluation of the migration ability of HCT116 cells, the sorafenib-treated group showed only a partial effect on the inhibition of migration; however, the CaLa-treated group showed almost the same effect on the inhibition of migration as in the combination group. The result of the quantitative analysis is provided in Supplementary Figure S1. Even if this was the case, the condition of the cells in the combination group was remarkably bad, with many floating and lysed cells found in morphological observation (Figure 4D).

## 3. Discussion

**Figure 5:**
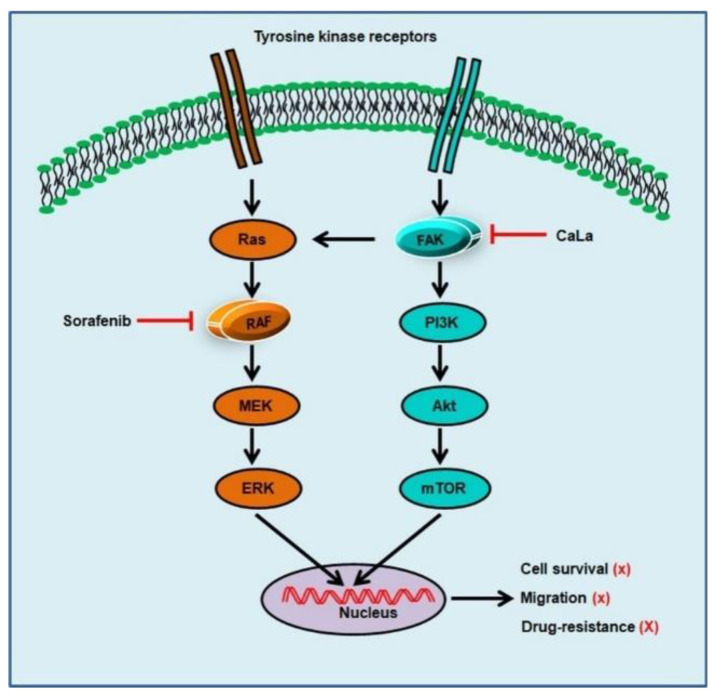
Schematic illustration of the signaling pathways that can be inhibited by sorafenib and calcium influx. Sorafenib inhibits the mitogen-activated protein kinase pathway following recombinant activated factor (RAF) inhibition, and calcium influx inhibits steroid receptor coactivator (Src)-mediated focal adhesion kinase (FAK), resulting in the inhibition of phosphoinositide 3-kinases (PI3K)/Akt/ mammalian target of rapamycin (mTOR) signaling.

In this study, we investigated whether sorafenib could show an enhanced anticancer effect against CRC cells following calcium-mediated inhibition of the bypassable survival signaling pathways of Src/FAK/Akt/mTOR. The anticancer efficacy of sorafenib was related to the inhibition of CRC cell growth through the inhibition of MAPK signal transduction via RAF downregulation, and enhanced antitumor effect of sorafenib was possible following the inhibition of sub-signals from FAK downregulation mediated by calcium influx (Figure 5).

CaLa is a crystalline salt formed by a reaction between lactic acid and calcium carbonate, and its neutral nature allows it to diffuse easily by penetrating the cell membrane; therefore, it was utilized for sustained calcium supply to CRC cells [12]. To observe the inhibitory effect of the FAK/Akt/mTOR pathways following calcium influx, the sensitivity of the phosphorylated form in signaling pathways was determined at the protein level. The Src family of protein tyrosine kinases is important in the regulation of growth and differentiation of cancer cells. Src activity is upregulated upon phosphorylation at Tyr^416^ in the kinase domain [14]. FAK is a non-receptor protein tyrosine kinase that acts as a substrate for Src and is a key element of integrin sub-signaling. FAK plays an important role in the cell proliferation, migration, cell death, and acceleration of the cell cycle in the G1 to S phase transition [15]. Tyr^397^ is the autophosphorylation site of FAK, and this phosphorylated site binds the SH2 domains of Src.

Akt, which is known as protein kinase B, is a ubiquitous kinase that plays an important role in diverse biological responses, such as cell survival and growth, by phosphorylating multiple proteins [16]. Akt promotes cell survival by inhibiting apoptosis, and the activation of Akt is dependent on a regulatory mechanism that requires its phosphorylation on Ser^473^ [17]. The active form of mTOR is determined by its phosphorylation at Ser^2448^ via the PI3K/Akt signaling pathway and it plays a key role in cell growth [18].

There are two potential reasons for the downregulation of these signaling molecules at the protein level. One is the downregulation of signaling cascades by enzymatic activation. The other is the collective enhancement of dephosphorylation at the active site belonging to signaling molecules. The continuous influx of calcium is the fundamental reason that makes the above two hypotheses possible because the existing studies have well established the role of various proteolytic and catabolic breakdown enzymes in the action of calcium, such as the activation of proteases and phosphatases [19,20]. However, proof of the concept related to enzymatic activation was not planned in the present study. Therefore, further studies will be carried out to examine a variety of possibilities related to the activity of calcium-based enzymes to provide a clear basis for calcium-dependent downregulation of the FAK signaling pathway.

Cell cycle arrest is the point of disruption in the cell cycle, where the cells are no longer involved in the processes surrounding division, and it is possible to observe signs of growth inhibition or cell death [21]. The G1 phase of the cell cycle is an intermediate phase occupying the time between the end of cell division in mitosis and the beginning of DNA replication [21,22]. Since the cells grow in preparation for DNA replication in this phase, cell growth could be suppressed under proliferation-quiescence through G1 arrest [22]. When CRC cells were treated with sorafenib, floating or lysed cells could not be observed in morphological analysis; therefore, cell cycle analysis was a good basis for supporting the mechanism of growth inhibition by sorafenib. Of course, a high concentration of sorafenib could induce other cell arrests; however, only G1 arrest was observed at low concentrations of sorafenib.

The increase in the sub-G1 accumulation of CRC cells was confirmed following treatment with CaLa. During apoptosis, genomic DNA is cleaved into smaller fragments [21]. This is a specific marker of apoptosis, for which propidium iodide-stained cells show a peak in the sub-G1 phase [21,22]. The appropriate CaLa concentration was applied for the combination study by fulfilling an equal screening method with sorafenib, and partial cell death was observed at selected concentrations. At this point, it must not be concluded that RAF is only responsible for cell growth and that FAK is only associated with cell survival. As it is impossible to determine the predominant signaling pathway between RAF and FAK in relation to the proliferation and survival of CRC cells, and RAF or FAK signaling pathways can complement each other for cancer cell proliferation and survival, the binary distinction must not be acceptable.

FAK is not only a critical modulator of signaling pathways mediated by tyrosine kinase receptors but also responds to stimuli from cell–cell adhesion proteins to control a variety of oncogenic cellular responses [11,16]. Src is a common intracellular point of convergence in the signaling initiated by integrin-extracellular matrix interactions [14]. The tyrosine kinase Src phosphorylates FAK, leading to increased FAK activity [11]. Further, the interaction between FAK and Src plays an important role in facilitating the intracellular signaling pathways for migration and invasion by inducing epithelial-mesenchymal transition [11,12,14].

FAK deactivation is considered to play a huge role in the calcium-mediated inhibition of migration, and combination with sorafenib could be a potential therapeutic strategy for the advanced stage of CRC. However, the significance of the calcium supply in the prevention or treatment of CRC is still controversial [23,24]. Therefore, it is necessary to establish a clear mode of action and reproducibility of the effect for clinical applications, and there is a need to additionally secure the applicable dosage and non-clinical toxicity.

In conclusion, our results indicated that calcium influx induced the deactivation of Src/FAK/Akt/mTOR, which is a bypassable signaling pathway of sorafenib. The combination of sorafenib with CaLa enhanced apoptosis and inhibited the metastatic features of CRC cells even when a lower dose of sorafenib was used. A clearer mode of action should be investigated in a future study with CRC cells that are completely unresponsive to sorafenib. Although the immediate clinical application may be premature, our findings indicate that the anticancer effect of sorafenib on patients with advanced CRC could be enhanced in combination with CaLa, if an appropriate usage/dosage is provided.

## 4. Materials and Methods

### 4.1. Cell Culture and Reagents

Human CRC cell lines (HCT-116 and HT-29) were purchased from American Type Culture Collection (Manassa, VA, USA). The cells were maintained in RPMI1640 medium (Welgene, Kyung-San, Korea) supplemented with 10% fetal bovine serum (Welgene), 10,000 units/mL penicillin, and 10,000 μg/mL streptomycin (Welgene) in a humidified atmosphere at 37 °C containing 5% CO_2_. Sorafenib [*N*-(3-trifluoromethyl-4-chlorophenyl)-*N*′-(4-(2-methyl carbamoyl pyridin-4-yl)oxyphenyl)urea] was synthesized at Bayer Corporation (West Haven, CT, USA), and CaLa was purchased from Sigma-Aldrich (St. Louis, MO, USA).

### 4.2. Cell Viability Assay

CRC cells were seeded at a density of 5000 cells/well in a 96-well plate and treated with the indicated concentrations of sorafenib and CaLa, alone and in combination. After 72 h, the medium was removed and 100 µL of 3-(4,5-dimethyl-2-thiazolyl)-2,5-diphenyl-2H-tetrazolium bromide (MTT, 5 mg/mL; Sigma-Aldrich) was added to each well, and the cells were incubated for 2 h at 37 °C in a humidified environment at 5% atmospheric CO_2_. The cells were then lysed, the formazan crystals formed were dissolved in 100 μL of dimethyl sulfoxide (DMSO, Sigma-Aldrich), and the absorbance was measured at 570 nm using an Epoch Micro-Volume spectrophotometer system (Bio-Tek, Winooski, VT, USA).

### 4.3. Cell Cycle Analysis

For cell cycle distribution analysis, 3 × 10^5^ CRC cells were seeded in 60 mm dishes and treated with CaLa and sorafenib, alone and in combination, for 72 h. After the treatment, the cells were collected through trypsinization (Sigma-Aldrich), centrifuged at 3000 rpm for 3 min at 4 °C, washed twice with ice-cold phosphate buffered saline (PBS, Welgene), and fixed with 70% cold ethanol (Sigma-Aldrich). The cells were then treated with 5 μL of a 10 mg/mL solution of ribonuclease and were stained with 10 μL of a 1 mg/mL solution of propidium iodide for 30 min at 37 °C in the dark (BD bioscience, Franklin Lakes, NJ, USA). The DNA content of the cells was measured using flow cytometry (BD FACSCalibur™, BD bioscience, NJ, USA), and the percentage of cells in each cell cycle phase was evaluated using BD FACS Comp^TM^ software (BD bioscience).

### 4.4. Western Blot Analysis

CRC cells were lysed using radioimmunoprecipitation assay (RIPA) lysis buffer (1% NP-40, 0.25% sodium deoxycholate, 50 mM Tris-HCl pH 7.4, 150 mM NaCl, 1 mM ethylenediaminetetraacetic acid (EDTA), 1% Triton X-100, 10% glycerol, 100 mM NaF, 1 mM NA3VO4, 10 mM NaPP, and a protease inhibitor cocktail (Roche, Basel, Switzerland)). The lysates were quantitated using a bicinchoninic acid assay kit (Thermo Scientific, Waltham, MA, USA) and were transferred to a polyvinylidene fluoride membrane (EMD Millipore, Billerica, MA, USA). After blocking in tris-buffered saline (TBS) with tween 20 (100 mM Tris-HCl, 1.5 M NaCl, 0.5% Tween-20, pH 7.5; Sigma-Aldrich) containing 5% non-fat milk (Biorad, CA, USA) for 1 h, the membranes were incubated overnight in a 4 °C cold room with a primary antibody at an appropriate dilution in TBS-T containing 5% BSA and 0.1% sodium azide (Sigma-Aldrich). The primary antibodies used in the study were: GAPDH (1:10,000, Cell Signaling, Danvers, MA, USA), pRAF (1:1000, Cell Signaling, Danvers, MA, USA), pERK (1:1000, Cell Signaling, Danvers, MA, USA), pSrc (1:1000, Cell Signaling, Danvers, MA, USA), pFAK (1:1000, Cell Signaling, Danvers, MA, USA), pAkt (1:1000, Cell Signaling, Danvers, MA, USA), and p-mTOR (1:1000, Cell Signaling, Danvers, MA, USA). After incubation, the membranes were incubated for 2 h with an anti-rabbit secondary antibody (1:5000, Cell Signaling, Danvers, MA, USA). The immunoblots were developed using western blotting detection reagents (Abclone, Seoul, Korea) and were exposed to an X-ray film (Agfa, Leverkusen, Germany) according to the manufacturer’s recommended protocol.

### 4.5. Intracellular Calcium Concentration

The cytosolic free calcium ion concentration was measured using a confocal laser scanning microscope (Leica, Heidelberg, Germany). Cultured HCT116 cells were loaded with 10 μM Fluo-3/AM and 1 μL of 25% Pluronic F-127 in dimethyl sulfoxide (Thermo Scientific, Waltham, MA, USA) and were incubated for 30 min at 37 °C. After loading the fluorescence probes, 2.5 mM CaLa was added and the intensity of green fluorescence was measured using Image J (imagej.nih.gov/ij).

### 4.6. Enzyme-Linked Immunosorbent Assay (ELISA)

The lysates from cultured CRC cells were concentrated using VIVASPIN^®^ (Sartorius Stedim Biotech, Goettingen, Germany). Quantitative analysis was performed using a human pSrc ELISA kit (Cell Signaling, Danvers, MA, USA) and human pFAK, pAkt, and p-mTOR ELISA kits (Invitrogen, Carlsbad, CA, USA) according to the manufacturer’s instructions.

### 4.7. Wound Healing Assay

For the wound healing assay, a culture insert consisting of two reservoirs separated by a 500 µm thick wall was used (ibidi, München, Germany). The CRC cells were seeded into a two-chamber (100 µL of 4 × 10^5^ cells/mL) culture insert (ibidi, München, Germany). After 24 h of incubation, the chamber was gently removed, creating a gap of ~500 µm. The cells were then allowed to migrate into the bare areas for 12 h. The cell imaging was performed using the JuLi Br Live-cell analyzer (NanoEnTek Inc., Guro-Dong, Korea).

### 4.8. Statistical Analysis

Data are presented as the mean ± standard deviation (SD). Statistical significance was analyzed using Student’s *t*-test or the Mann–Whitney rank-sum test depending on the normality of the data. A difference of *p* < 0.05 was considered statistically significant. All statistical analyses were performed using Sigma Stat ver. 3.5 (Systat Software Inc., San Jose, CA, USA).

## Supplementary Materials

The following are available online, Figure S1: Quantitative analysis for scratching wound healing assay, Figure S2: Cell morphology following sorafenib treatment in colorectal cancer (CRC) cells, Figure S3: Cell morphology following lactate calcium salt (CaLa) treatment in colorectal cancer cells, Figure S4: Cell morphology following combination treatment in colorectal cancer (CRC) cells.
