# Supplementary material for: Combination Antitumor Effect of Sorafenib via Calcium-Dependent Deactivation of Focal Adhesion Kinase Targeting Colorectal Cancer Cells
Source: Molecules. 2020 Nov 13;25(22):5299. doi: 10.3390/molecules25225299 (PMC7697278; doi:10.3390/molecules25225299)
Supplement: Supplementary file 1 [file molecules-25-05299-s001.pdf]

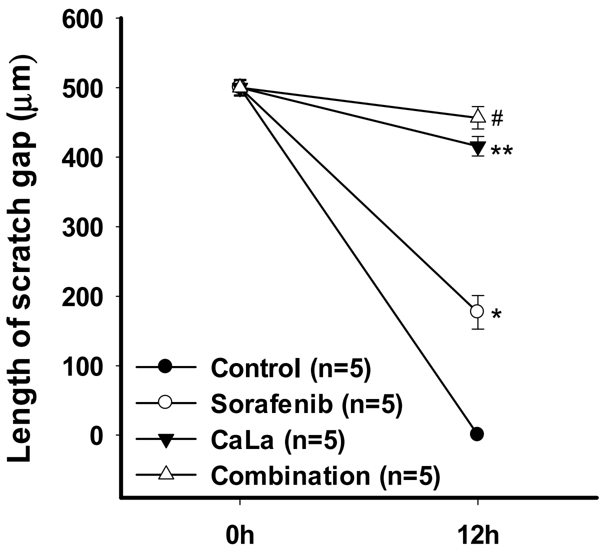

Figure S1. Quantitative analysis for scratching wound healing assay. *P<0.05 and **P<0.001 vs. Control; #P<0.001 vs Control, Sorafenib, and CaLa. Results are mean ± S.D.

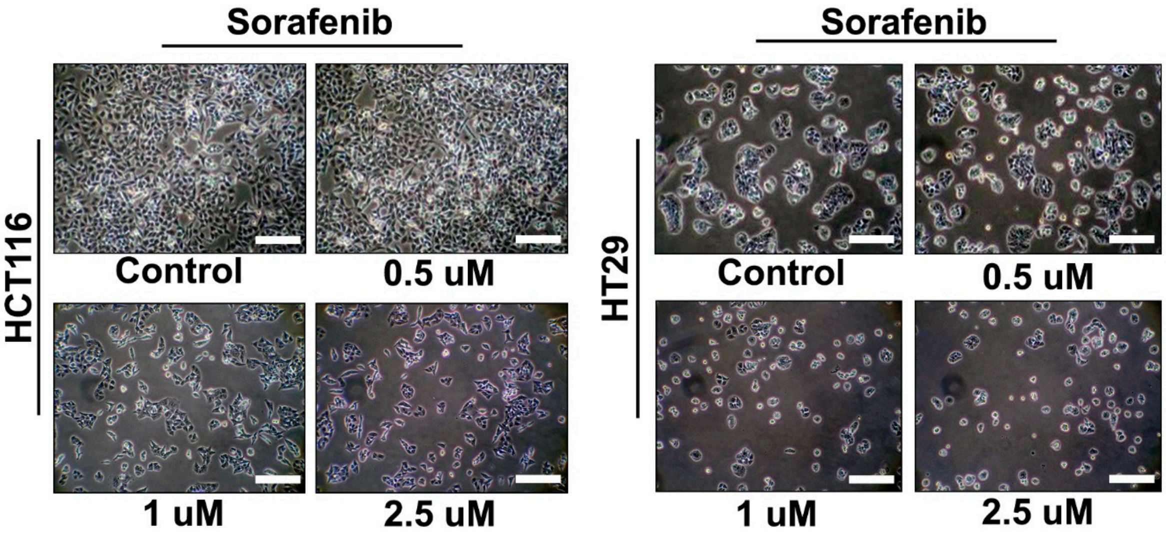

Figure S2. Cell morphology following sorafenib treatment on colorectal cancer cells. Scale bars: 100 μm.

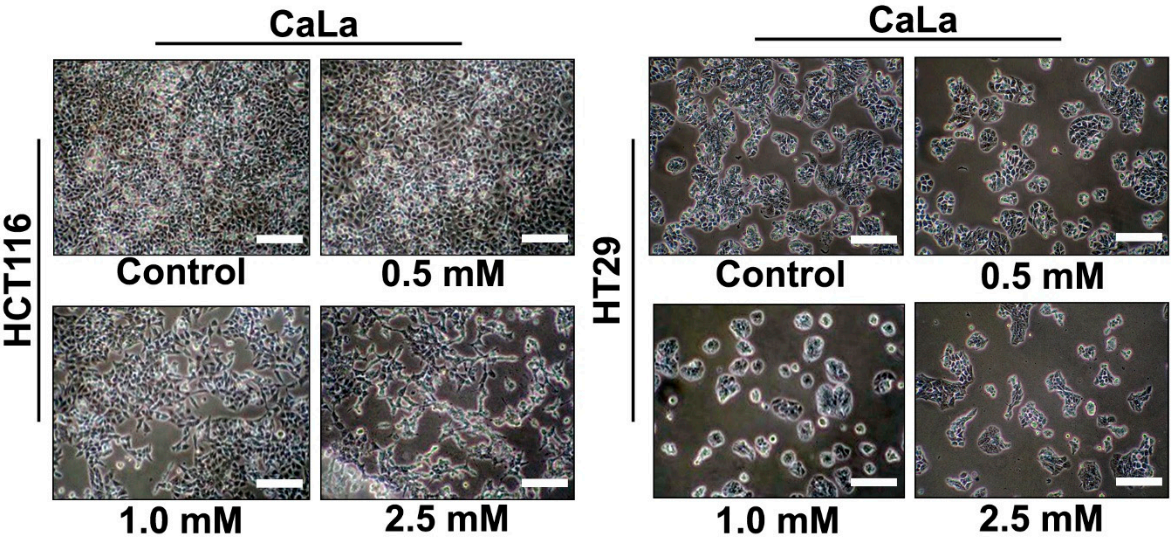

Figure S3. Cell morphology following lactate calcium salt (CaLa) treatment on colorectal cancer cells. Scale bars: 100 μm.

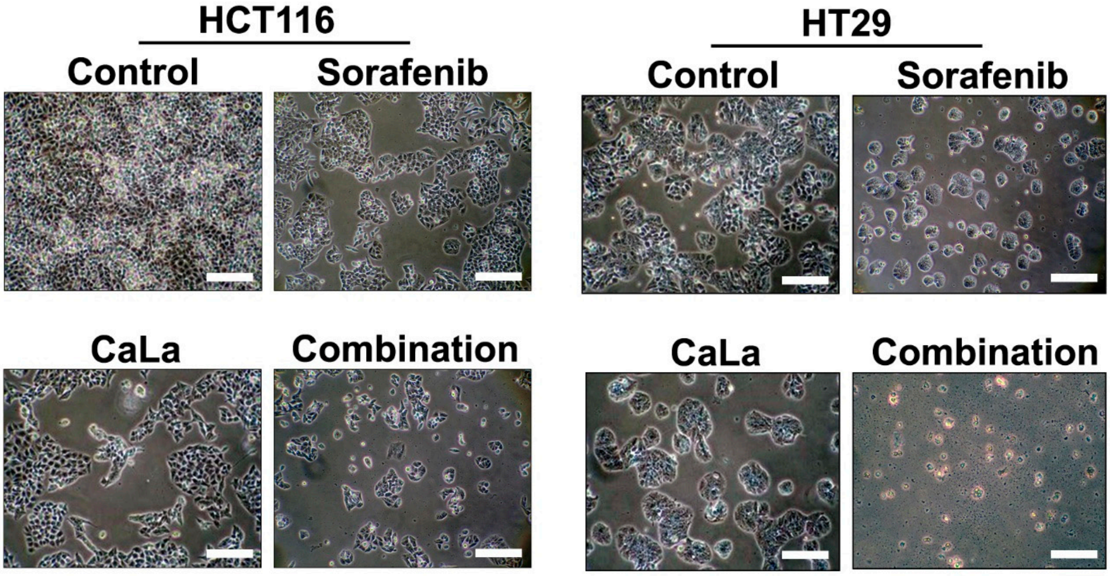

Figure S4. Cell morphology following combination treatment on colorectal cancer cells. Scale bars: 100 μm. CaLa: lactate calcium salt.
